# Intellectual Disability Associated With Pyridoxine-Responsive Epilepsies: The Need to Protect Cognitive Development

**DOI:** 10.3389/fpsyt.2019.00116

**Published:** 2019-03-08

**Authors:** Bjørnar Hassel, Ane Gretesdatter Rogne, Sigrun Hope

**Affiliations:** ^1^Department for Neurohabilitation, Oslo University Hospital and University of Oslo, Oslo, Norway; ^2^Norwegian Defence Research Establishment (FFI), Kjeller, Norway

**Keywords:** vitamin B6, pyridoxine-responsive epilepsy, intellectual disability, lysine metabolism, α-aminoadipic semialdehyde, γ-glutamic semialdehyde, aldehydes, hyperprolinemia type II

## Abstract

Pyridoxine (vitamin B6)-responsive epilepsies are severe forms of epilepsy that manifest as seizures immediately after birth, sometimes *in utero*, sometimes months, or years after birth. Seizures may be treated efficiently by life-long supplementation with pyridoxine or its biologically active form, pyridoxal phosphate, but even so patients may become intellectually disabled, for which there currently is no effective treatment. The condition may be caused by mutations in several genes (*TNSALP, PIGV, PIGL, PIGO, PNPO, PROSC, ALDH7A1, MOCS2*, or *ALDH4A1*). Mutations in *ALDH7A1, MOCS2*, and *ALDH4A1* entail build-up of reactive aldehydes (α-aminoadipic semialdehyde, γ-glutamic semialdehyde) that may react non-enzymatically with macromolecules of brain cells. Such reactions may alter the function of macromolecules, and they may produce “advanced glycation end products” (AGEs). AGEs trigger inflammation in the brain. This understanding points to aldehyde-quenching, anti-AGE, or anti-inflammatory therapies as possible strategies to protect cognitive development and prevent intellectual disability in affected children. Studies on how aldehydes traverse cell membranes and how they affect brain function could further the development of therapies for patients with pyridoxine-responsive epilepsies.

## Introduction

### Pyridoxine-Responsive Epilepsy: Clinical Manifestations and Underlying Molecular Causes

Pyridoxine-responsive epilepsy is a severe form of epilepsy that manifests as generalized seizures immediately after birth, sometimes *in utero*. In some patients seizures begin some months or years after birth (1, 2). Life-long treatment with high doses of pyridoxine (vitamin B6) or its derivative pyridoxal 5′-phosphate (PLP) is efficient with respect to the epileptic seizures; withdrawal of pyridoxine may precipitate life-threatening *status epilepticus*, which is only reversed when pyridoxine is reinstated. Conventional antiepileptic drugs probably have no place in the treatment of this disorder. Pyridoxine-responsive epilepsy may entail intellectual disability and other neurodevelopmental impairments, such as attention deficit/hyperactivity disorder and autism (3, 4), which are not prevented by pyridoxine treatment alone, pointing to separate mechanisms underlying the epilepsy and the cognitive impairment. Only in some cases is the metabolic disturbance accompanied by structural changes (white matter changes, hippocampal sclerosis) that may contribute to the epilepsy or the intellectual disability.

## Pyridoxine: The Biologically Active Form and its Entry Into the Brain

To be biologically active, pyridoxine must be converted to PLP. This two-step conversion involves phosphorylation of pyridoxine to pyridoxine 5′-phosphate, which is then oxidized to PLP. PLP is a co-enzyme in more than 100 biochemical reactions, many involving amino acid metabolism (5). With respect to brain function, PLP is essential for the formation of various neurotransmitters and neuromodulators that are amino acids or derived from amino acids: monoamines, GABA, glutamate, glycine, D-serine, and taurine (5, 6). Through its involvement in amino acid metabolism PLP is necessary for energy production in all brain cells.

**Figure 1:**
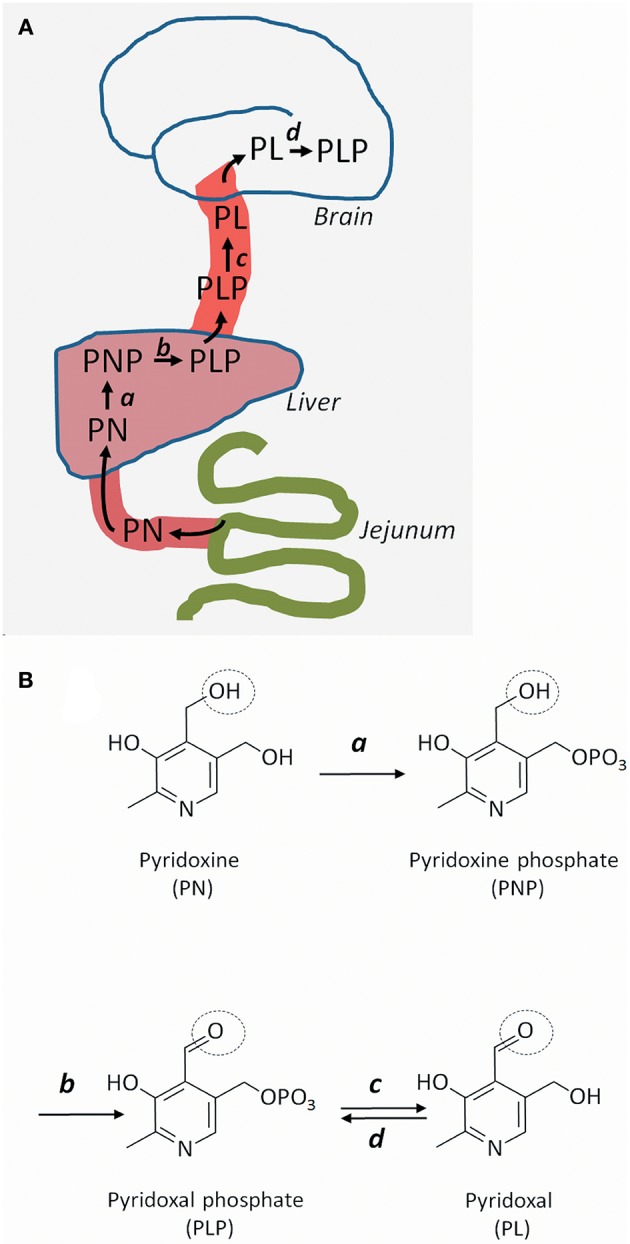
Uptake, transport, and metabolism of pyridoxine and its derivatives. **(A)** Pyridoxine (PN) (or its closely related form pyridoxamine), is transported from the gut to the liver, where it is metabolized successively to pyridoxine phosphate (PNP) and pyridoxal phosphate (PLP). PLP is given off to the circulation, but cannot cross the blood-brain barrier and enter the brain unless it is converted to pyridoxal (PL). Once inside brain cells, PL is re-phosphorylated to PLP. **(B)** Molecular structures of pyridoxine and its metabolites. PN is phosphorylated to PNP by pyridoxine kinase (a) in the liver. PNP is oxidized to PLP by PNP oxidase (b). In brain capillaries, PLP is dephosphorylated to PL by tissue non-specific alkaline phosphatase (c). In brain cells, PL is re-phosphorylated to PLP by PL kinase (d). The circle shows the OH group that gives rise to the aldehyde function in PLP and PL.

Pyridoxine's route from food ingredient to active coenzyme in the brain is somewhat complex. Pyridoxine is taken up in the gut and converted into PLP in the liver (Figures 1A,B). PLP is given off to the circulation, but for PLP to enter the brain, it has to lose its phosphate group and become pyridoxal. This dephosphorylation (from PLP to pyridoxal) is performed by an extracellular enzyme: tissue non-specific alkaline phosphatase, which is attached to cell surfaces by glycosylphosphatidylinositol (GPI) anchors. Once inside brain cells, pyridoxal is reconverted to PLP by the enzyme pyridoxal kinase (7). Reduced expression of pyridoxal kinase in mice leads to low levels of PLP in the brain and severe seizures (8); a similar condition has not yet been reported in humans.

## Genetic Causes of Pyridoxine-Responsive Epilepsy: Some Affect Metabolism of Pyridoxine, Some Lead to Build-up of Reactive Metabolites That Inactivate PLP

Pyridoxine-responsive epilepsy is caused by rare, mostly recessive loss-of-function mutations in various genes. Some mutations affect the metabolism of pyridoxine, whereas some lead to accumulation of reactive metabolites that inactivate PLP (Table 1). The *PNPO* gene encodes the enzyme pyridoxine 5′-phosphate oxidase, which is rate-limiting for the formation of PLP in the liver (9) (Figure 1). Mutations affect the enzymes' ability to bind its cofactor (flavin mononucleotide) and its interaction with PLP-dependent enzymes (10). The result is reduced formation of PLP, a low circulating level of PLP, and a low availability of PLP (i.e., pyridoxal) to the brain. This deficiency may be amended with administration of PLP rather than pyridoxine.

**Table 1:** Genes whose mutations cause defective PLP metabolism or inactivation of PLP through accumulation of reactive compounds.

	**Gene**	**Protein**	**Protein function**	**Effect of mutation**
Mutations related to PLP metabolism	*PNPO*	Pyridoxine phosphate oxidase	PLP synthesis from PNP	Lack of PLP in liver and hence in blood
	*TNSALP*	Tissue non-specific alkaline phosphatase	PL formation from PLP	Lack of PL formed at the blood-brain barrier
	*PIGV, PIGL, PIGO*	GPI-anchors	Anchoring tissue non-specific alkaline phosphatase at capillaries	Lack of PL formed at the blood-brain barrier
	*PROSC*	Proline synthase cotranscribed, aka “Pyridoxal phosphate homeostasis protein”	Cellular PLP homeostasis?	Increased cellular PLP consumption?
	*PDXK*	Pyridoxal kinase	PLP synthesis	Lack of PLP in cells (only in knock-out mouse model)
Mutations that cause PLP inactivation	*ALDH7A1*	α-Aminoadipic semialdehyde dehydrogenase	α-Aminoadipate	Accumulation of α-aminoadipic semialdehyde and P6C, which inactivates PLP
	*MOCS2*	Molybdenum cofactor synthesis 2	Involved in oxidation of sulphite to sulfate	Accumulation of sulphite, which inactivates α-aminoadipic semialdehyde dehydrogenase, causing accumulation of α-aminoadipic semialdehyde and P6C, which inactivates PLP
	*ALDH4A1*	P5C dehydrogenase	Metabolism of proline	Accumulation of γ-glutamic semialdehyde and P5C, which inactivates PLP

*All mutations cause epilepsy, which (except in the case of experimental pyridoxal kinase deficiency) respond to pyridoxine therapy. GPI, glycosylphosphatidylinositol; PLP, pyridoxal phosphate; PL, pyridoxal; PNP, pyridoxine phosphate; P6C, L-Δ^1^-piperidine-6-carboxylate; P5C, Δ^1^-pyrroline-5-carboxylate*.

The *TNSALP* gene encodes tissue non-specific alkaline phosphatase, which is present at the blood-brain barrier, among other tissues. This phosphatase dephosphorylates blood-borne PLP to pyridoxal, which may enter the brain. Loss-of-function *TNSALP* mutations lead to ineffective PLP dephosphorylation to pyridoxal, reduced pyridoxal transfer into the brain, and accumulation of PLP in blood (11). Pyridoxine treatment alleviates the ensuing epilepsy (12) probably by raising the serum level of PLP even further to achieve some increase in pyridoxal level. This approach may be effective, because the mechanism for transport of pyridoxal across the blood-brain barrier is not saturated, so that any increase in serum pyridoxal concentration will lead to increased uptake into the brain (13). The disorder, which is termed hypophosphatasia due to the low circulating level of alkaline phosphatase, may be treated with enzyme replacement therapy (14), which reduces the need for pyridoxine therapy (12).

Several proteins are involved in the formation of GPI anchors that tether extracellular tissue non-specific alkaline phosphatase to the cell membrane. Mutations in some genes (*PIGV, PIGL, PIGO*) (15, 16) that encode GPI-related proteins are known to cause pyridoxine-responsive epilepsy (17). Suboptimal GPI anchoring of tissue non-specific alkaline phosphatase at the blood-brain barrier probably explains the pyridoxine-responsiveness of the epilepsy of these patients (18). The dysfunctional anchoring of alkaline phosphatase is also the reason for the high circulating level of alkaline phosphatase in this disorder known as hyperphosphatasia or Mabry syndrome.

The *ALDH7A1* gene encodes the enzyme α-aminoadipic semialdehyde dehydrogenase (aka antiquitin), which is involved in lysine metabolism. The mutation, the incidence of which has recently been estimated at 1.6:100 000 (19), causes build-up of the lysine metabolite α-aminoadipic semialdehyde, its cyclic form L-Δ^1^-piperidine-6-carboxylate (P6C), and their common precursor L-pipecolic acid. The level of these metabolites in plasma, urine, and cerebrospinal fluid (CSF) is typically elevated with *ALDH7A1* mutations (20, 21). P6C has been found to bind and inactivate PLP (22). This inactivation is thought to underlie the pyridoxine responsiveness of the seizures in this condition, which is often termed “pyridoxine-*dependent* epilepsy.” A similar situation is caused by *MOCS2* mutations, which lead to molybdenum cofactor deficiency and sulphite accumulation. Sulphite has been shown to inhibit α-aminoadipic semialdehyde dehydrogenase (23), causing build-up of α-aminoadipic semialdehyde and hence of P6C, which inactivates PLP (24). In hyperprolinemia type II, mutations in *ALDH4A1* (Δ^1^-pyrroline-5-carboxylate dehydrogenase) lead to accumulation of γ-glutamic semialdehyde and its cyclic form pyrroline-5-carboxylic acid (P5C). P5C reacts with PLP in a manner similar to P6C (25) to produces PLP deficiency in the brain (26).

The *PROSC* gene encodes a cytosolic protein that binds PLP. The function of the protein remains unknown, but it has been suggested to act as an intracellular PLP reservoir, preventing PLP, itself a reactive aldehyde, to react spontaneously with other cell constituents (27). *PROSC* mutations entail low CSF levels of PLP, possibly because PLP is consumed through spontaneous reactions with other cell constituents, and the epilepsy that accompanies the condition is therefore pyridoxine-responsive.

Thus, it seems that a lack of PLP in the brain is the cause of epilepsy in all the above-mentioned conditions: in *TNSALP* mutations and in mutations causing dysfunctional GPI anchoring of tissue non-specific alkaline phosphatase the transfer of PLP in the form of pyridoxal into the brain is suboptimal; in *PNPO* mutations, PLP is not produced normally in the liver; in *ALDH7A1, MOCS2*, and *ALDH4A1* mutations, PLP is inactivated by accumulating reactive metabolites. In *PROSC* mutations PLP may be consumed as it acts as a reactive metabolite toward other cell constituents (Table 1). In all cases treatment with pyridoxine or PLP itself alleviates the seizure tendency (2).

It has been hypothesized that a deficiency of PLP may cause seizures through suboptimal inhibitory GABAergic neurotransmission. However, in a study on one patient with pyridoxine-responsive epilepsy (reported before the genetic origin had been discovered) the CSF obtained by lumbar puncture had a level of glutamate that was 200 times higher than normal, indicative of dysfunctional glutamate metabolism, but the level of GABA was normal (28). With pyridoxine treatment the CSF level of glutamate was normalized, and that of GABA remained normal. Realizing that PLP is essential in more than 100 enzymatic reactions (5), ascribing pyridoxine-responsive seizures to one enzymatic dysfunction may seem reductionist. Intriguingly, in four patients with hyperprolinemia type II and pyridoxine-responsive epilepsy serum PLP was normal (29), and in two other cases of pyridoxine-responsive epilepsy, the level of PLP in CSF was normal (28, 30). The latter observation could suggest that CSF levels of PLP do not reliably reflect intracellular PLP levels in brain cells (which do not accumulate PLP, but pyridoxal) (13), or that the biochemistry of the mutations is more complex than one would expect from the (hitherto) known functions of the genes.

## Intellectual Disability in Pyridoxine-Responsive Epilepsy: The Need for Novel Therapies

Pyridoxine-responsive epilepsy is often associated with intellectual disability (2, 4, 10, 27, 29, 31). The intellectual disability is not prevented by pyridoxine treatment alone, and patients tend to be intellectually disabled although their epilepsy is well controlled. These observations point to unique mechanisms underlying the intellectual disability. Some effects of the metabolic derangement may be unspecific, such as lesions to periventricular white matter or to the hippocampus in the form of hippocampal sclerosis (27, 32), and such structural damage could play a role in the intellectual disability in addition to the biochemical alteration. In many cases, however, magnetic resonance imaging (MRI) of the brain is normal, and the cause of intellectual disability must be sought elsewhere.

Intellectual disability is diagnosed when a person has an IQ score below 70, when he/she has impaired adaptive skills, and when the disability was manifest before the person reached 18 years of age (33). Intellectual disability implies that development of complex brain functions, or cognitive functions, is slowed or arrested (34). Intellectual disability encompasses impairment in abstract thinking, language, numerical understanding, problem solving, and learning i.e., intellectual skills. It further implies impairment in executive functioning: the ability to initiate, prioritize, plan, and carry out actions, and the ability to regulate one's own emotions and impulses, and it affects the understanding of social cues and rules. Another area of disability is visuospatial understanding, or the comprehension of space and movement, manifesting as clumsiness in the execution of practical work and problem solving. Intellectual disability leaves the affected person with low psychomotor speed and limited attention span and working memory, which lead to slow information processing and vulnerability to distraction and fatigability. The degree of disability ranges from mild, through moderate and severe, to profound. Regardless of degree, the all-encompassing nature of intellectual disability renders the disabled person at an enormous disadvantage in managing his/her own adult life in an independent manner. Therefore, identification of ways to ameliorate or compensate for metabolic dysfunctions that entail intellectual disability is sorely needed. However, research into molecular mechanisms that may form the basis for therapy is scant. Below we therefore point to possibilities that arise from the understanding of the reactivity of the metabolites that accumulate in some of the pyridoxine-responsive epilepsies.

## Possible Toxic Mechanisms of α-Aminoadipic Semialdehyde, γ-Glutamic Semialdehyde, P6C, and P5C. Therapeutic Possibilities

Several mutations that cause pyridoxine-responsive epilepsy lead to accumulation of reactive compounds: α-aminoadipic semialdehyde and P6C in *ALDH7A1* and *MOCS2* mutations (20, 23), γ-glutamyl semialdehyde and P5C in *ALDH4A1* mutations (25). These compounds must be assumed to accumulate in brain cells, and they emerge as likely pathogenic factors underlying the intellectual disability that accompanies pyridoxine-responsive epilepsy. Their levels would not be expected to decrease with pyridoxine therapy. An important mechanistic observation has come from intervention studies on patients with *ALDH7A1* mutations. These patients seem to improve cognitively on a diet that is poor in lysine, but enriched in arginine (32). The dose of dietary arginine was 150 mg arginine/kg bodyweight/day or higher in children that were from 288 days to 8 years of age (32). Arginine supplementation probably inhibits transfer of lysine across the blood-brain barrier, as the two amino acids travel competitively on the same transporter (35). The net result would be a reduction of the exposure of the brain to lysine, pointing to lysine metabolites (α-aminoadipic semialdehyde and its derivatives) as an important cause of intellectual disability in in *ALDH7A1* mutations. It cannot be ruled out, however, that such dietary measures may have biological effects in themselves that are not related to the effects of *ALDH7A1* mutations. Arginine supplementation has a number of biological effects in humans (36, 37), some of which may be related to arginine's role as a precursor for nitric oxide.

α-Aminoadipic semialdehyde and γ-glutamic semialdehyde may, because of their aldehyde groups, react non-enzymatically with proteins and probably other macromolecules, such as DNA, RNA and phospholipids, and with glutathione and other –SH-group-containing molecules (38–40). The reaction of proteins and other molecules with an aldehyde may change their function or turnover. Such reactions further lead to the formation of “advanced glycation end products” (AGEs). Other endogenous aldehydes, such as glucose in its open chain form (41), methylglyoxal (42), glyceraldehyde (43–45), and acetaldehyde (46), are known to participate in the formation of AGEs, a reactivity that is an important aspect of their pathogenicity. AGEs may trigger pathological inflammation reactions through their activation of AGE receptors [RAGEs; (47)]. This mechanism has been proposed to contribute to the cognitive derangement in Alzheimer disease and in the neuronal dysfunction observed in diabetes (44, 48). At present, a pathogenic role of spontaneous reactions between aldehydes and other cell constituents is only hypothetical. Even so, two possible therapeutic strategies emerge in the subset of pyridoxine-responsive epilepsies that are associated with aldehyde accumulation: prevention of AGE formation and inhibition of the downstream inflammatory reactions that AGEs may precipitate.

Prevention of AGE formation or inactivation of AGEs has proved possible with both metformin (49), a commonly used antidiabetic drug, and with trans-resveratrol, a polyphenol found in berries, and hesperetin, a flavonoid found in citrus fruit (50). Some studies suggest an anti-AGE effect of pyridoxine itself (51–53), while others do not confirm this (54–56). It may be possible to scavenge α-aminoadipic semialdehyde and γ-glutamic semialdehyde by other means as well, e.g., by increasing the availability of free SH groups in the form of glutathione or N-acetyl-cysteine. The reactivity of α-aminoadipic semialdehyde and γ-glutamic semialdehyde toward free –SH groups has not been determined, however. Treatments or diets that reduce the total exposure of the brain to AGE-forming compounds (e.g., from glucose, fructose, or their metabolites glyceraldehyde and methylglyoxal) may be beneficial when the level of α-aminoadipic semialdehyde or γ-glutamic semialdehyde is high. Such a diet may be one that keeps serum glucose low (57). The downstream inflammatory response to AGE formation may possibly be targeted by anti-inflammatory drugs, scavengers of reactive oxygen species, and RAGE antagonists (58–60).

The cyclic forms of α-aminoadipic semialdehyde, P6C, and γ-glutamic semialdehyde, P5C, are reactive toward PLP (22, 25), probably reflecting a general reactivity that could lead these compounds to modify other metabolites or cell constituents as well. However, this has not yet been studied in much detail.

It is not known where in the brain α-aminoadipic semialdehyde and γ-glutamic semialdehyde may exert their (supposed) toxic action, for instance, whether it occurs intra- or extracellularly. One missing factor that is necessary for the understanding of the toxicity of aldehydes is knowledge of how aldehydes cross cell membranes in the brain. From toxicological and metabolic studies (42, 61) it is clear that various aldehydes do cross cell membranes, but the transfer mechanism remains unknown and therefore escapes therapeutic manipulation.

## A Pathogenic Lack of Downstream Products During Enzyme Dysfunction?

Although accumulation of toxic metabolites may be a primary mechanism of pathology in enzyme deficiencies, it cannot be ruled out that reduced formation of downstream metabolites contributes to the overall phenotype. In *ALDH7A1* mutation, α-aminoadipic semialdehyde is not converted into α-aminoadipate (and hence α-ketoadipate) at a normal rate. α-Aminoadipate may have important physiological functions as it may act as a ligand at the NMDA-type glutamate receptor, and it may modulate kynurenate metabolism (62). Further, α-aminoadipate is noted for its fairly selectively toxic effects on glial cells (63), which may be a reflection of a hitherto unexplored physiological effect of α-aminoadipate at lower concentrations. Therefore, a lack of α-aminoadipic semialdehyde dehydrogenase function may have biological consequences caused by downstream effect as well as by the accumulation of aldehyde substrate. A recently developed *aldh7a1* knock-out zebra fish (64) could be a suitable model for the testing of therapeutic effects of α-aminoadipate in loss of α-aminoadipic semialdehyde dehydrogenase function.

## Caveats in the Treatment of Pyridoxine-Responsive Epilepsies and Associated Intellectual Disability

The use of pyridoxine at high doses must take into account that pyridoxine itself may be neurotoxic in the long run, causing polyneuropathy (65, 66), for which patients should be monitored. Further, the use of a diet that is rich in arginine to reduce transport of lysine across the blood-brain barrier (31) may lead to higher levels of γ-glutamic semialdehyde (and P5C), an arginine metabolite, that may lead to AGE formation. Lastly, the use of scavengers to reduce tissue concentration of α-aminoadipic semialdehyde in *ALDH7A1* mutations or γ-glutamic semialdehyde in *ALDH4A1* mutations must take into account that the scavenger might react with PLP itself, reducing the effectiveness of pyridoxine treatment.

The published reference values for PLP in CSF obtained through lumbar puncture cover a fairly narrow concentration range: 11–46 nmol/L in adults (67), 32–89 nmol/L (68), or 23–64 nmol/L (29) in children. In a recent study, we used HPLC and tandem mass spectrometry to measure PLP and other pyridoxine metabolites in CSF from the cerebral ventricles of 15 patients undergoing CSF drainage because of raised intracranial pressure (69); at the time of sampling the intracranial pressure was normal, and the CSF was clear and colorless. The range of PLP values in ventricular CSF was great: 3.65–132 nmol/L (median 11.3 nmol/L); pyridoxal range was 6.4–51 nmol/L (median 20 nmol/L); the range for pyridoxic acid, the metabolic product of pyridoxal, was 0.32–14.5 (median 1.77 nmol/L). Similar findings for PLP were reported by Footitt et al. (70). Thus, with a 36-fold difference between the lowest and the highest PLP value, it may be that the natural variation in CSF levels of PLP may explain differences in pyridoxine requirement among patients, and it may explain why some patients develop intellectual disability and others do not.

With respect to dietary treatment of patients suffering from *ALDH7A1* mutations with lysine restriction and arginine supplementation it should be realized that results on intellectual disability are preliminary, and that a better understanding of the variation in clinical response as well as possible side effects of the treatment is needed.

## Conclusion

Pyridoxine-responsive epilepsies are often compounded by neurodevelopmental disorders, most importantly intellectual disability. The epilepsies may be treated efficiently with pyridoxine or PLP, but the intellectual disability does not respond to such treatment. In some forms of pyridoxine-responsive epilepsy the underlying mutation entails accumulation of certain aldehydes that may react non-enzymatically with brain cells constituents, altering their function. Alternatively, the aldehydes may, after binding to proteins and forming AGEs, produce inflammation upon activation of AGE receptors. Dietary measures to reduce the brain's exposure to lysine metabolite α-aminoadipic semialdehyde in *ALDH7A1* mutations has shown promise with respect to ameliorating intellectual disability; this was achieved by reducing dietary lysine and by dietary arginine supplementation upwards of 150 mg arginine/kg bodyweight/day in children that were from 288 days to 8 years of age (32).

Future therapies aimed at protecting cognitive development in affected children may exploit the possibility of inactivating the accumulating aldehydes, inhibiting AGE formation or AGE receptors, or dampening the inflammatory response that follows from AGE receptor activation.

## Author Contributions

All authors listed have made a substantial, direct and intellectual contribution to the work, and approved it for publication.

### Conflict of Interest Statement

The authors declare that the research was conducted in the absence of any commercial or financial relationships that could be construed as a potential conflict of interest.

## Abbreviations

AGE: advanced glycation end product
CSF: cerebrospinal fluid
GABA: γ-amino butyric acid
GPI anchors: glycosylphosphatidylinositol anchors
MRI: magnetic resonance imaging
PLP: pyridoxal 5′-phosphate
P5C: Δ^1^-pyrroline-5-carboxylate
P6C: L-Δ^1^-piperidine-6-carboxylate
RAGE: receptor for advanced glycation end products.
